# Prophylactic treatments of migraine and GPs in the north of France: evaluation of practices

**DOI:** 10.1186/1129-2377-1-S14-P213

**Published:** 2013-02-21

**Authors:** CL Christian

**Affiliations:** 1 Service de Neurologie et Pathologie Neurovasculaire, France

## Introduction

Prophylactic treatments of migraine are an important part of the management of the disease. Only one survey concerning this topic was performed in 2000 in France among GPs. This survey was however performed before the French migraine guidelines with a large use of dihydroergotamine at this time.

## Purpose

To evaluate the practices of GPS in the North of France concerning the prophylactic treatments of migraine management and to compare them with the French Guidelines.

## Methods

A self-administered questionnaire concerning prophylactic treatments of migraine was mailed in 2011 to 307 GPs in two big cities in the North of France (Lille and Roubaix). We analysed the data and compared them with the French Guidelines.

## Results

142 GPs answered to the questionnaire (46.2 %). 85% of GPs use prophylactic treatments of migraine when the patient has 4 to 5 migraine attacks per month. The first line treatment are beta-bloquers (BB) (60%). The first objective is to reduce the migraine attacks frequency by 50% at 3 months for 53% of the GPs and the second to increase the quality of life in 45%. 59% of GPs prescribe prophylactic treatment of migraine for a 6-12 months duration.

## Discussion

GPS in the North of France take into account the bad quality of life of migrainers to start a prophylactic treatment. They use in majority the recommended prophylactic treatments of migraine and during a correct duration according the French Guidelines.

## Conclusion

There is a dramatic increase since 2000 of the use of BB in France as first line prophylactic treatment of migraine. French guidelines of migraine seems to be useful for GPs.
